# Is Distant Extension Always Upset? Neural Evidence of Empathy and Brand Association Affect Distant Extension Evaluation

**DOI:** 10.3389/fpsyg.2022.804797

**Published:** 2022-02-01

**Authors:** Zhijie Song, Chang Liu, Rui Shi, Kunpeng Jing

**Affiliations:** School of Economics and Management, Yanshan University, Qinhuangdao, China

**Keywords:** distant extension, brand association, empathy, event-related potentials, corporate social responsibility, competence

## Abstract

Distant brand extension as an essential strategy of obtaining benefits was highly focused on the normal marketing practice and academic research. In the current study, we aim to recognize that how individuals with different levels of empathy respond to distant extensions under corporate social responsibility (CSR) and corporate competence (CC) associations to explore the corresponding neural mechanisms using event-related potentials (ERPs). We divided subjects into two groups involving a high empathy (HE) group and a low empathy (LE) group according to an empathy measure questionnaire. The subjects first faced a brand name following the CSR or CC association descriptions, and then, they were asked to evaluate the new product of brand by a five-point scale. Current results revealed that the participants of the HE group were more apt to accept the distant extension products than those of the LE group. Additionally, in the HE group, products from a brand with CSR associations were more acceptable than CC associations. Moreover, a larger N2 amplitude was elicited in the LE group than in the HE group. For the LE group, an augment N2 was found under CSR than CC associations, reflecting that LE consumers might perceive conflict when evaluating distant extensions and allocate more cognitive resources to deal with CSR information. At the later stage, the HE group showed a greater P3 than the LE group. For the HE group, an increased P3 was elicited under CSR than CC associations, suggesting that empathic individuals might show motivational salience and helping willingness toward distant extension products, especially under the CSR scenario. These results provide potential electrophysiological evidence for the positive impact of brand associations on the evaluation of distant brand extension in the case of subdividing different empathic individuals.

## Introduction

Brand extension, whereby a well-established brand uses its name to launch new products not offered ever before, is a beneficial and popular branding strategy (Keller and Aaker, 1992; Swaminathan, 2003). It is frequently used by modern enterprises in a realistic marketplace. To expand corporate market share, multiple established brands endeavor to extend their products into new categories relatively dissimilar with their core business range, namely, distant brand extension (Su et al., 2021). For instance, Apple successfully extended from phones to watches, Ferrari went beyond sports cars to introduce perfume, and Nestle went from human’s coffee, tea, and milk powder to pets’ food and drinks. Reflecting this trend, a growing stream of literature has focused on adopting appropriate strategies to introduce incongruent new products for benefiting from the parental brand equity, mostly (Jhang et al., 2012; Zhong et al., 2020; Gerrath and Biraglia, 2021). Several scholars identified some factors, such as strong brand reputation (Chun et al., 2015), products displayed placement (Zheng et al., 2019), and life-role transition of consumers (Su et al., 2021) to promote distant brand extensions success. In fact, despite the strategy of distant extensions having a degree of risk (Loken et al., 2002), from a long-term profit perspective, it can also gain much more potential benefits, such as expanding the financial revenue stream, dispersing sales pressure, and grabbing market resources (Parker et al., 2017). As a result, it is critical to understand how to enhance the positive evaluation of consumers toward distant extensions to reduce the risk of increasing enterprise costs.

Brand association, which is usually considered as the perception, cognitive, affect, and else information grasping of a person toward a company (Brown and Dacin, 1997), plays a significant role in a new product evaluation (Berens et al., 2005; Rubio and Marin, 2015). A strong and unique brand association benefits the brand extension evaluation (Keller, 1993). Generally, two essential types of brand associations were highly mentioned by previous researchers, that is, corporate competence (CC) association and corporate social responsibility (CSR) association. Specifically, CC association is commonly seen as perceptions of consumers about the ability of parent brand, such as superior technology, innovational competence, whereas the construction of CSR association is often characterized by a brand with high efforts to critical societal issues, such as engaging in social welfare, donation, and support activities (Brown and Dacin, 1997). For the two types of brand associations, previous scholars held different insights about its effect on brand extension evaluation. For example, Johnson et al. (2019) considered that CSR associations are generally seen as more valuable when consumers evaluate distant extensions than CC associations because they provide warm perceptions for consumers, while CC associations are not thought to. Inversely, Wang and Liu (2020) deemed that CC associations, providing direct evidence of product qualities, can help consumers deliver competence-related attributes of the parent brand to an extension product and further give a better extension evaluation. Notably, previous neuroimaging evidence has provided an insight that different types of brand association messages are processed diversely in brain activities of consumers, reflecting different mental processes among various consumer segments (Chen et al., 2015). In particular, a recent functional MRI (fMRI) study conducted by Medina et al. (2021) demonstrated that the brain response to the processing of CSR messages between low and high social awareness consumers was distinct. Accordingly, although the two types of associations positively influence product evaluations of brands, we suspect that individuals with different prosocial levels do not respond equally toward all CC and CSR associations (Lee and Cho, 2018; Xue et al., 2020). Further, it may be that the individual prosocial differences may result in distinctions in distant extension evaluations in the context of CC and CSR associations.

Empathy, as an essential prosocial capability, pertains to the human ability to understand and experience other emotions, reliably measured by psychometric scales, such as the Empathy Quotient (EQ) scale (Davis, 1983; Lawrence et al., 2004). It encompasses two components of affective (closely related to sympathetic emotions) and cognitive (focused on the attributed mental states of others) (Davis, 1994). The empathy-altruism hypothesis implies that the empathic response is tightly linked with prosocial behaviors (Batson and Shaw, 1991; Pelligra and Vásquez, 2020). Based on the hypothesis, the emotional mechanism and altruistic attributes of empathy obtained great attention from previous studies. For instance, prior evidence has shown that individuals with high empathy levels can be easily evoked empathy responses. They can perform a series of social support behavior, such as donating to charity (Lee et al., 2014), assisting unfortunate persons (Tangney et al., 2007), and caring for others’ pain (Flasbeck and Brüne, 2019). Furthermore, this kind of support behavior takes place not only among human beings but also can be motivated when consumers evaluate brands or products. To be specific, subjects with high empathy showed more preference for vulnerable brands (Kraus et al., 2012) or increased willingness to pay for fair-trade products (Zerbini et al., 2019). Contrarily, individuals with low empathy are self-interest orientation and usually care for their own well-being (Cialdini et al., 1997). From the perspective of neuroscience, Lee (2016) adopted electroencephalogram (EEG) measurement to uncover the neural mechanism of consumer empathy in response to corporate social responsibility messages and found that high empathy individuals are more inclined to pay for prosocial brand products than low empathy ones. Recently, He et al. (2021b) employed the functional near-infrared spectroscopy (fNIRS) technique to capture brain activations of subjects when they viewed advertising and demonstrated that empathic connections could increase positive attitudes of consumers toward advertising evaluation. However, in terms of brand extension evaluation, how individuals with varying levels of empathy respond to distant extensions under diverse brand associations, in particular, their neural mechanism, is unknown.

Neuroscientific tools, as novel and efficient measurement, can help capture automatic and implicit processing of subjects to solve these issues (Solnais et al., 2013). These approaches can overcome the limitations of traditional methods (e.g., questionnaire surveys and face-to-face interviews) that are short of objectivity and rarely better understand cognitive and affective processes of subjects (He et al., 2021a; Pei and Li, 2021). In the consumer neuroscience realm, multiple neurological techniques were used by previous scholars to explore the neural mechanisms underlying the brand extension evaluation processing. For example, an fMRI approach was employed to reveal the connection between the corresponding brain regions and emotional processing during the extension evaluation (Yang et al., 2021). By contrast, event-related potentials (ERPs), owing to the characters of low cost and high temporal resolution (milliseconds), have been extensively applied (Ma et al., 2007, 2008, 2014, 2020, 2021; Wang et al., 2012; Fudali-Czyz et al., 2016; Shang et al., 2017; Yang et al., 2018; Song et al., 2020). By using this method, some of the studies have identified a variety of valuable components to investigate the cognitive process of consumers during brand extension evaluation, such as the perceived conflict (N270) (Ma et al., 2007), categorization process (P300 and N400) (Ma et al., 2008; Wang et al., 2012), or affect transfer effect (LPP) (Song et al., 2020).

Specifically, the typical N2 component is a negative-going potential with a time window of approximately 200–400 ms at the frontal areas (Donkers and Van Boxtel, 2004; Folstein and Petten, 2008; Yoder and Decety, 2014). Multiple previous evidence has shown that N2 is sensitive to mismatch and conflict-related monitoring toward the stimuli (Veen and Carter, 2002; Larson et al., 2014; Han et al., 2015). For instance, Shang et al. (2017) indicated that a greater N2 amplitude was responded to products presented with social risk sentences than the control condition, reflecting stronger cognitive and emotional conflict under social interactions. The authors explained that consumers might need to regulate the cognitive conflict between their own purchase desire and the incongruence information from social communications, which was reflected by the larger N2 amplitude. Moreover, in brand extension research, higher N2 amplitudes were recorded for distant extensions rather than near extensions, reflecting perceived risk and conflict between the original brand and extensions (Ma et al., 2007, 2020; Song et al., 2020). Compared with previous studies, the stimuli of extension products were all relatively far from the original brands in this study. Although the given brands have good associations, the conflict effect of the brand extension might emerge in the current study. Furthermore, it has been suggested that positive emotions of consumers could reduce the cognitive conflict, in which case a decreased N2 amplitude was observed (Jin et al., 2018). Compared with low empathy individuals, empathic consumers were more apt to be elicited positive affect responses by good brand performance (Lee, 2016). Meanwhile, consumers with different prosocial traits respond in a dissimilar way toward CSR and CC brand association messages (Chen et al., 2015; Medina et al., 2021). Accordingly, we hypothesize that different N2 amplitude would be evoked between high empathy and low empathy groups.

A P3 component is a positive-going wave over the central to parietal regions with a peak latency range of 300–500 ms after stimulus onset (Polich and Kok, 1995). It has been indicated that the P3 component was linked to processes of stimuli categorization evaluation (Azizian et al., 2006). Previous studies investigating the neural mechanism of brand extension evaluation have demonstrated that an enhanced P3 was observed by category congruence between the original brands and extension products (Ma et al., 2008; Shang et al., 2017). In addition, the P3 component can also be elicited by affective/motivational stimuli with higher amplitudes (Nieuwenhuis et al., 2005; Jin et al., 2020). More importantly, neuromarketing research has found that P3 was sensitive to the prosocial motivation and helping behaviors (Chiu Loke et al., 2011; Carlson et al., 2016; Teng et al., 2018). For example, Chiu Loke et al. (2011) indicated that women who scored higher in the Prosocialness Scale were more inclined to make helping decisions than men, which was reflected by larger P3 amplitude. Carlson et al. (2016) revealed that the P3 component could predict prosocial motivation and behaviors of participants, and a notable P3 amplitude was found for evaluating high-empathy targets than low-empathy targets. Recently, Liu et al. (2021) examined the different brain activities when evaluating the distant extension products with different brand reputations, which found the CSR condition elicited larger LPP (P3 family) amplitude than the ability reputation condition. Based on the abovementioned research, because CSR messages are more prosocial and empathetic, we posit that larger P3 amplitudes would be observed in CSR than CC associations and in HE than the LE group.

In total, the N2 and P3 components were applied to investigate the neural distinctions among different empathy individuals toward distant extension products under two types of brand associations. In the experiment, participants were first divided into two groups according to individual scores of an empathy questionnaire used by previous studies. During the ERP measurement, each of them was successively presented to a series of procedures: a brand name following an association activity (CSR or CC), after that, EEG was recorded while participants evaluated the new product of brands. Following recent research by Liao et al. (2019) and Liu et al. (2021), to better understand, two fixed verbs were applied for connecting the above target stimuli (brand, association activity, and product name). Finally, a five-item scale ranging from 1 to 5 was applied to investigate the consumers’ acceptance of distant extensions.

## Materials and Methods

### Participants

In the current experiment, 42 Chinese native speakers, including undergraduate and graduate students from Yanshan University, were recruited. All the participants did not have any psychiatric illness or mental disorders. They were all healthy, right-handed, and had a normal or corrected-to-normal vision. Prior to the EEG recording, a written informed consent was obtained from every participant. This study was approved by the institutional review board. Data from two participants were excluded due to technical problems and excessive EEG artifacts. Therefore, the final sample included data for a total of 40 participants (19 women). The age of them ranged from 18 to 33 years (*M* = 24.36 years, *SD* = 3.74 years). They were paid 30 yuan (around USD 13) for taking part in the experiment.

### Stimulus Design

The dairy brand was viewed as an appropriate choice, as most Chinese consumers, the subject group type used in the experiment, mostly drink milk daily, with a high focus on brand development (Ozdemir et al., 2020). Accordingly, five well-known national Chinese dairy brands (Yili, Mengniu, Wangzai, Sanyuan, and Guangming) were selected as the brand stimuli materials according to the ranking list on chinapp.com. The five brands mainly run business in dairy products, such as milk beverages, with no difference on social responsibility and competence performed in daily life. Besides, none of them has been exposed to scandal in terms of lacking social responsibilities or capabilities recently. Prior to the experiment, all the participants self-reported that they were all familiar with the given five brands, such as the brand name and product attribute information. In addition, the clothes category is not belonging to the main business scale of dairy brands, which was considered a relatively distant extension category from the original beverage brands by previous studies (Ma et al., 2007). Thus, five clothes products (e.g., t-shirts) were selected to the present product stimuli. Significantly, to date, these dairy brands have not been extended to the clothes industries in the Chinese marketplace.

For the brand association activity construct, according to Johnson et al. (2017), in which the CC association highlighted the characteristics of enterprises through high product quality communications, as well as the CSR association mainly emphasized its social welfare. Thus, a total of 12 brand association descriptions of CC (*n* = 6) and CSR (*n* = 6) were generated by a discussion group consisting of four marketing doctors. Every description of brand association was limited to the four Chinese characters. Next, a group of subjects (*n* = 60) who did not engage in the formal experiment and the former discussion group was invited to rate the appropriateness of brand association descriptions through an online survey. A 5-point Likert scale ranging from 1 to 5 (“1 = strongly disagree” and “5 = strongly agree.”) was used for all item responses. Finally, eight brand association descriptions were determined. For the four CC descriptions: technology development (*M* = 6.112, *SD* = 1.024), technological innovation (*M* = 6.022, *SD* = 1.112), technical communication (*M* = 5.634, *SD* = 1.014), and quality improvement (*M* = 5.600, *SD* = 1.001); for the four CSR descriptions: targeted poverty alleviation (*M* = 6.532, *SD* = 1.201), education of children (*M* = 6.201, *SD* = 1.021), charitable donation (*M* = 5.955, *SD* = 1.029), and rural vitalization (*M* = 5.543, *SD* = 1.002).

### Procedure

A 2 (empathy level: high, low) × 2 (brand association: corporate social responsibility and corporate competence) between-subjects design was conducted in the experiment. For the empathy level measurement, the present study referenced Lee (2016) and Yen and Yang (2018) to design a 7-point Likert scale containing six items. To ensure the effectiveness of the scale, we followed the cross-cultural adaptation procedure (Beaton et al., 2000). First, the English empathy scale was initially translated into a Chinese version by two Chinese doctors majoring in neuromarketing. Then, another translator translated it back into English and compared the consistency between the two versions. Next, two professors proofread the descriptions of all measurement items to ensure the construct validity. Finally, the scale was tested again on the basis of a pilot study toward 10 Chinese consumers. All above participants reported that the Chinese version of the empathy scale was clear and suitable.

To categorize participant groups, a sample of 120 students (60 men mean age = 21.5 years, *SD* = 3.5 years) majoring in marketing were invited to fill in the empathy scale approximately 2 weeks before the ERPs experiment. We selected the participants according to the average scores of the five items falling above 82.5% or below 17.5% of the whole sample. In the end, a total of 42 students with the extreme average scores in the empathy scale (Chronbach’s alpha = 0.90) were divided into the HE group (*N* = 21, 13 women) and LE group (*N* = 21, 14 men). They were all willing to take part in the EEG measures of our experiment.

**FIGURE 1:**
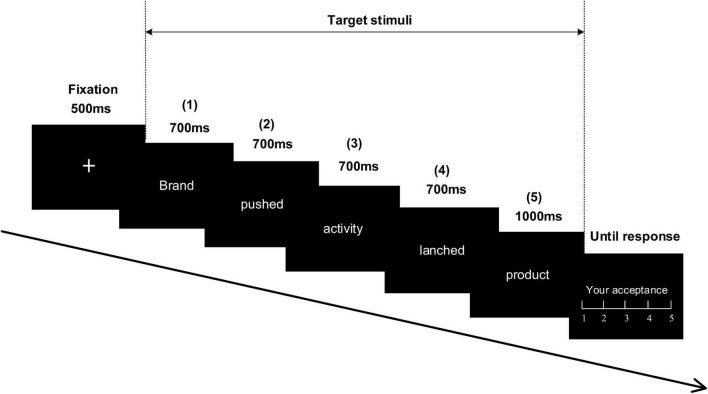
Experimental procedure: participants were successively presented to a series of stimuli of which the five target stimuli formed as one sentence in Chinese. They were asked to press a button (1–5) based on their acceptance level toward the new product of a brand.

The participants were performed to sit in a dim and sound-attenuated room at a viewing distance of 70 cm from a computer screen. All the stimuli were presented in the center of a computer-controlled monitor (1,024 × 768 pixels, 60 Hz). Based on a recent work by Liu et al. (2021), each group of stimuli in the current study employed the formation of a sentence. Specifically, two fixed verbs were adopted to connect among the brand name, activity, and product name, consisting of the five parts: brand (1) + pushed (2) + association activity (3) + launched (4) + product (5) (e.g., “Yili pushed technological innovation and launched T-shirt” or “Yili pushed charitable donation and launched T-shirt”). And each part of the stimuli group was successively presented in Chinese characters (white and bold song font, size 30). They were all controlled with a range of two to four characters with a visual angle of 2.1–4.5 degrees × 0.8 degrees and positioned at the center of the black screen. As shown in Figure 1, a fixation cross displayed at the beginning of each trial for 500 ms on a blank screen, prompting the start of a target trial. Next, 1–4 parts of the target stimulus appeared for 700 ms, followed by a blank screen for 500 ms. In addition, there was a random interval, respectively, lasting for 500–800 ms among them, following a product name (the 5 part) was subsequently shown for 1,000 ms. Finally, after a black screen appeared for 500 ms, a five-point Likert scale was presented for participants to rate the acceptance degree toward the brand extension from 1 “very unacceptable” to 5 “very acceptable” by pressing a button on a mini keypad. The rating scale disappeared immediately on the screen when they gave feedback. Before the formal experiment, all participants were provided six stimulus groups to practice. Totally, 200 stimuli groups (each consisting of 1–5 parts) were involved in the experiment and pseudo-randomly assigned into four blocks, with each block holding 50 trials. The E-Prime 2.0 software (Psychology Software Tools, Pittsburgh, PA, United States) was used for the stimuli presented and behavioral button recordings. The experiment totally lasted for approximately 35 min.

### Electroencephalography Recordings and Analyses

The electroencephalography data were collected from 64 Ag/AgCl electrodes placed on an electrode cap arranged in the international 10–20 system with a Brain actiCHamp amplifier (Brain Products GmbH, Munich, Germany). The electrode impendence during the recording was maintained below 10 kΩ. The sampling rate was 500 Hz, and the online bandpass filter was between 0.05 and 100 Hz. The vertical and horizontal electrooculogram was measured by two electrodes placed supraorbital and infraorbital location on the left and right eye and the two electrodes placed laterally at the outer canthi of both eyes. The scalp EEGs were online referenced to the Cz site and offline referenced the average of the TP9 (left mastoid) and TP10 (right mastoid) during recording.

The BrainVision Analyzer 2.1 software was used to analyze the offline EEG data. The data were filtered using a low-pass filter at 30 Hz (24 dB/Octave). According to Semlitsch et al. (1986), the independent component analysis (ICA) method was applied for correcting the artifacts (e.g., eye blink). The filtered EEG data were segmented into an 800 ms epoch surrounding the trigger with 200 ms before the stimuli onset of the product name presentation as a baseline. Any trials exceeding ± 100 V were rejected from the calculating. The EEG data are referenced to the average of all the electrodes. The EEG epochs were averaged separately for two groups of participants with the two association conditions (HE-CSR, HE-CC, LE-CSR, and LE-CC). On the basis of the visual observations of our data, N2 and P3 components were analyzed. For the N2, nine electrodes were selected (F1, Fz, F2, FC1, FCz, FC2, C1, Cz, and C2), following previous studies (Jing et al., 2019; Zhang et al., 2019), within the time window of 260–320 ms. For the P3, nine electrodes (C1, Cz, C2, CP1, CPz, CP2, P1, Pz, and P2) were selected over the central-parietal area (Xie et al., 2016; Tang et al., 2021), within the time window of 350–450 ms. Repeated-measured ANOVAs were calculated for behavioral data and ERP data. If there was any interaction effect, a simple effect analysis was conducted. The Greenhouse–Geisser correction (Greenhouse and Geisser, 1959) was applied to correct the sphericity assumption violations. Spearman’s correlation analyses were conducted between the empathy score of consumers and the acceptance level (AL) of brand extension, as well as between the P3 amplitude and the AL.

## Results

### Behavioral Results

**FIGURE 2:**
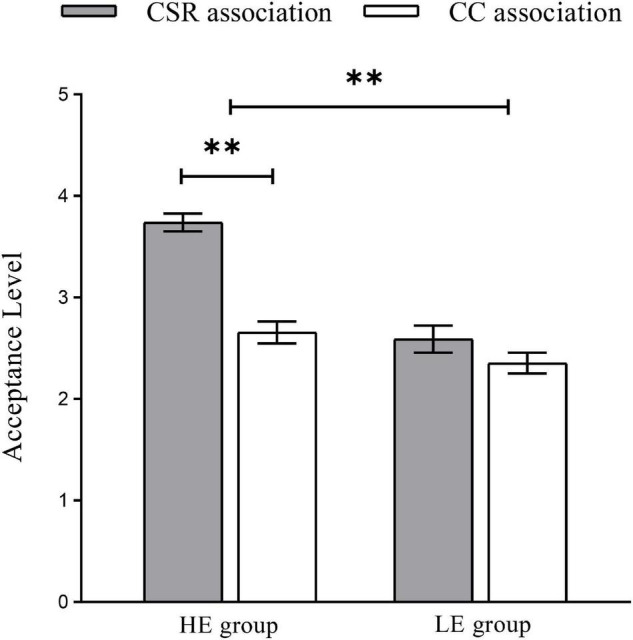
The acceptance level of distant extension products under the corporate social responsibility (CSR) or corporate competence (CC) associations in HE and LE groups. ^**^p < 0.01.

We performed a 2 (high empathy group vs. low empathy group) × 2 (corporate social responsibility association vs. corporate competence association) ANOVAs to analyze the reaction time (RT) and AL. For the RT, no significant main effect and interactive effect were observed (*p* > 0.05). For the AL, the main effect of the empathy group [*F*(1, 19) = 65.109, *p* < 0.001, η2 *p* = 0.774] and brand association [*F*(1, 19) = 54.194, *p* < 0.001, η2 *p* = 0.740], were significant. Besides, the AL for the HE group (*M* = 3.198, *SE* = 0.073) and CSR association (*M* = 3.166, *SE* = 0.081) was higher than LE group (*M* = 2.472, *SE* = 0.077) and brand CC association (*M* = 2.504, *SE* = 0.068), respectively. The interaction effect between the two empathy groups was notable [*F*(1, 19) = 10.765, *p* < 0.05, η2 *p* = 0.362]. Thus, we conducted a simple effect analysis to evaluate the interactive effects (as shown in Figure 2). For the HE group, the difference between the CSR and CC association was significant [*F*(1, 19) = 68.430, *p* < 0.001, η2 *p* = 0.783], indicating that consumers were more willing to accept the distant extension products when consumers perceived a brand with CSR association (*M* = 3.740, *SE* = 0.086) than CC association (*M* = 2.656, *SE* = 0.109). But for the LE group, no significant effect was found between the CSR and CC association (*p* > 0.05). Spearman’s correlation analyses showed that the empathy score of consumers was positively related to the AL (*r* = 0.573, *p* < 0.001).

### Event-Related Potential Results

#### N2 (260–320 ms)

**FIGURE 3:**
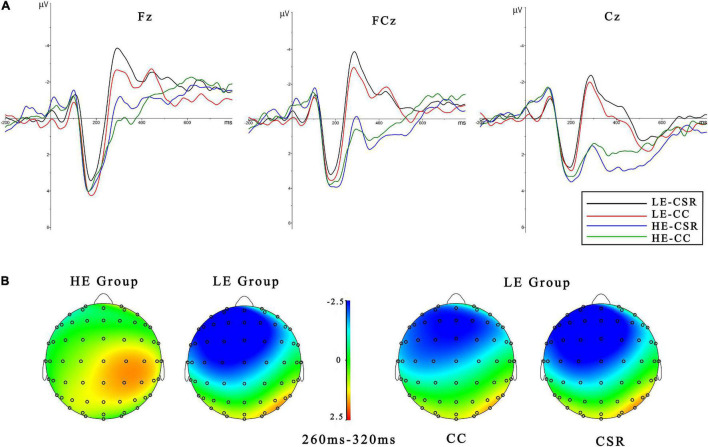
Event-related potentials (ERPs) results. **(A)** Grand averaged waveforms of N2 for high empathy (HE) and low empathy (LE) groups under the two conditions of CSR and CC associations at midline electrodes (Fz, FCz, and Cz). **(B)** The topographic maps for different conditions with the window of 260–320 ms.

A 2 × 2 × 9 electrode ANOVA was conducted for N2. As shown in Figure 3, the results revealed a significant effect of the empathy group [*F*(1, 19) = 18.922, *p* < 0.001, η2 *p* = 0.499], the brand association [*F*(1, 19) = 4.636, *p* < 0.05, η2 *p* = 0.196], and the electrode [*F*(1, 19) = 3.386, *p* < 0.05, η2 *p* = 0.693]. The LE group (*M* = − 2.457 μV, *SE* = 0.466) elicited a slightly larger N2 than the HE group (*M* = 0.803 μV, *SE* = 0.646). For the electrode distribution, the left sits distribute in F1, FC1, and C1 (*M* = − 0.935 μV, *SE* = 0.240) and the midline sites distribute in Fz, FCz, and Cz (*M* = − 0.936 μV, *SE* = 0.281) were larger than the right sites (*M* = − 0.583 μV, *SE* = 0.297). Meanwhile, there was a trend of brand association effect [*F*(1, 19) = 3.351, *p* = 0.083, η2 *p* = 150] in LE group. The CSR association (*M* = − 2.791 μV, *SE* = 0.490) elicited a larger N2 than the CC one (*M* = − 2.123 μV, *SE* = 0.511). But no brand association effect was found in HE group (*p* > 0.1).

#### P3 (350–450 ms)

**FIGURE 4:**
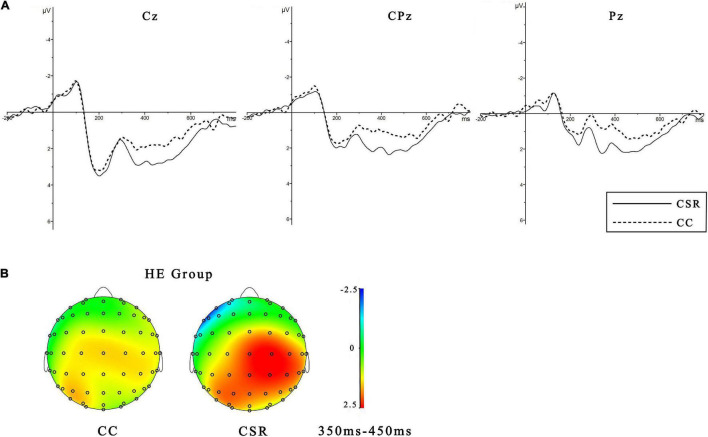
Event-related potentials results. **(A)** Grand averaged waveforms of P3 for HE groups under the two conditions of CSR and CC associations at midline electrodes (Cz, CPz, and Pz). **(B)** The topographic maps for different conditions with the time window of 350–450 ms.

The 2 × 2 × 9 ANOVA revealed a main effect of the empathy group [*F*(1, 19) = 5.146, *p* < 0.05, η2 *p* = 0.213]. The HE group (*M* = 1.629 μV, *SE* = 0.479) elicited a significantly larger P3 than did the LE group (*M* = 0.096 μV, *SE* = 0.420). The interaction effect between the empathy group and brand association [*F*(1, 19) = 12.921, *p* < 0.05, η2 *p* = 0.405] was notable. The simple effect analysis found that there was a significant effect of brand association [*F*(1, 19) = 4.688, *p* < 0.05, η2 *p* = 0.198] in HE group (as shown in Figure 4). Further, the CSR association (*M* = 2.028 μV, *SE* = 0.566) elicited a larger P300 than the CC one (*M* = 1.229 μV, *SE* = 0.454). But for LE group, no significant brand association effect was found (*p* > 0.1). Spearman’s correlation analyses revealed that the P3 amplitudes on Cz (*r* = 0.431, *p* < 0.001), C2 (*r* = 0.451, *p* < 0.001), CP1 (*r* = 0.312, *p* < 0.01), and CP2 (*r* = 0.329, *p* < 0.01) were positively related to the AL.

## Discussion

The present study takes a new perspective by focusing on the factors of individual characteristics differences and brand associations influencing the brand extension evaluation. Since enterprises launching new category products can help extend their business scale, it is necessary to identify the potential factors that affect the evaluation of distant extension products and improve their success (Parker et al., 2017). Thus, this study aims to assess whether EEG analyses differ in the neural processing of the distant extension evaluations under CSR and CC associations. Further, considering the distinctions in individuals, we subdivided consumers into two levels of empathy, HE, and LE, to explore the issue.

With respect to the behavioral data, the AL toward distant extension products was higher in the HE group than in the LE group. The higher the empathy score of consumers was, the more they accepted the distant extensions. Previous studies have demonstrated that individuals who scored in high empathy are more inclined to make supporting behaviors toward brands (Kraus et al., 2012). In fact, new products of brands obtaining positive evaluation can help enterprises successfully broaden the product business scale and revenue streams. It may be that HE consumers were more apt to give a positive evaluation toward new products with good brand associations for supporting favorable brands. However, LE consumers, owing to their self-interest orientation, might not elicit good feelings when brands hold positive associations. Instead, they would consider the suitable level between the original brand and distant extension products from a personal perspective. Further, it is possible that LE consumers would not positively evaluate distant extensions for the reason of brands holding good associations. As for brand association, the AL was higher under CSR than CC association, exclusively in the HE group. Social responsibility associations mean a brand with prosocial traits, kindness, and helpfulness, while competence associations with capabilities, skillfulness, creativity, and high-quality signals (Brown and Dacin, 1997; Berens et al., 2007). Compared with CC associations, the orientation of CSR information is more congruent with HE consumers who focus positively on other- or social-related welfare (Johnson et al., 2017). Thereby, HE consumers might tend to positively evaluate the new products under CSR associations, even though they belong to a distinct category from their original brand products.

For the neural level, the N2 amplitude was greater in the LE group than in the HE group. Previous studies have demonstrated that N2 is an early component indicator of conflict detection or cognitive control (Folstein and Petten, 2008). The higher the conflict between the presented stimuli, the larger N2 amplitude was observed. In particular, in brand extension evaluation research, dissimilar extension products, due to their incongruence with their original brands, were elicited by an increased N2 than near extension products. Since the strategy of distant brand extensions was usually seen as illogical or impossible, it could recruit potential risk for original brands. The result of the current study is probably because the LE group might engage more cognitive resources in response to the distant brand extension at an early stage than the HE group. Specifically, participants low on empathy might objectively evaluate extended products from a rational perspective due to the much consideration of their own well-being. Thus, they may perceive greater risk and conflicts toward the extension products even though the given brands hold positive associations. Conversely, for individuals high on empathy, which are commonly characterized as emotional, a brand with good associations could elicit their positive affect. There is evidence that a positive emotion could decrease the conflict perception toward brand products, as reflected by a smaller N2 (Jin et al., 2018). In the current study, HE individuals might transfer the good affective toward brands to the extension products, which further lightens the conflict perception of distant extension products.

Moreover, a recent neuroimaging research verified that when consumers with characterizing as self-promotion or low social awareness processed CSR messages, brain areas associated with emotion regulation were strongly triggered (Medina et al., 2021). They explained that the type of self-orientation of consumers might lighten and regulate the potential positive emotions caused by CSR information. Similarly, in the present study, a stronger N2 amplitude was activated by CSR rather than CC information in the LE group. In fact, compared with underlying self-competence of enterprises, CSR activities primarily fulfill social-related obligations to benefit others. It may be that consumers with low empathy levels are usually focusing on the personal well-being, whose orientation does not fit the other-oriented goal of CSR context. Thus, they might need more cognitive resources to regulate the mismatch and conflict between their self-interest goal and the altruistic goal orientation of corporates.

Regarding the P3 component, previous brand extension research has revealed that enhanced P3 amplitudes were linked with category similarity (Ma et al., 2008; Fudali-Czyz et al., 2016; Shang et al., 2017) or impossibility target detection (Yang et al., 2018). Unlike those studies, the extension product stimuli were far from the original brands in the current study. Meanwhile, we observed that an enlarged P3 was elicited by the HE group than the LE group. Notably, prior studies have also shown that P3 was sensitive to motivational/affective salience stimuli and prosocial behavior, especially among humans with prosocial traits (Chiu Loke et al., 2011). In our experiment, the stimuli shown for participants were brands with good associations extending to new products. It has been reported that good associations could help consumers form a favorite brand impression (Keller, 1993). As mentioned above, the higher the empathy level of consumers is, the more acceptance they evaluate the distant extensions under positive brand associations. Compared with individuals with LE levels, empathic consumers are prosocial and generally apt to help persons or brands when they need assistance (Zerbini et al., 2019). Previous studies have found that the larger P3 amplitude is positively associated with more helping behaviors (Carlson et al., 2016). In line with previous studies, we observed a positive correlation between the P3 amplitude and behavioral responses of consumers. Specifically, the larger the P3 amplitudes were, the more the consumers were willing to accept the distant extensions. In fact, the aim of brands launching distant extension products is usually for expanding the business scale of company. In other words, the brands need support and assistance of consumers when launching distant extension products. In this study, HE consumers might hold stronger helping motivation, and thus, they would be more inclined to support favorable brands to launch new products than LE consumers.

In addition, a recent ERPs study has revealed that messages of corporate social responsibility and ability are processed differently in the human brain. To be specific, a CSR brand evoked greater LPP (P3 family) amplitude than ability descriptions when evaluating distant extensions, which suggested that consumers might hold stronger altruistic motivation and assistance intension for new products of a CSR brand. Similarly, in this study, we observed an enhanced P3 amplitude in response to CSR than CC associations, but only in the HE group. Compared with CC associations, the presentation of CSR messages represents helpful value to others (Peloza and Shang, 2010). Previous neural evidence has shown that high empathetic individuals are more willing to assist others whose personal values are similar to themselves (Masten et al., 2011). In the current study, one possible explanation is that HE consumers might be more apt to support the extension products from a brand with CSR associations due to their consistent value of other orientation. Furthermore, as the empathy-altruism hypothesis suggested, empathic individuals are more sensitive to social and emotional information and are commonly driven by altruistic motivation (Pelligra and Vásquez, 2020). Accordingly, the result of P3 could be explained by the augmented motivational salience and helping willingness when HE participants evaluate extension products from a brand with CSR associations.

To recap, the results from this study show that consumers who hold different levels of empathy respond to a brand with CSR and CC associations in different brain regions while evaluating distant extension products. Briefly, the HE group elicited decreased N2 and enhanced P3 amplitudes than the LE group. Moreover, larger N2 amplitudes in the LE group and P3 in the HE group were, respectively, observed under CSR than CC associations. The results might reflect that empathic participants allocate less cognitive conflict and stronger helpful motivation toward favorable brands when evaluating distant extensions. This context differs from prior brand extension research that assisted the value growth of brands *via* launching products of a near category to ensure success. From a new perspective, this study focuses on adopting appropriate brand association strategies to introduce extensions increasingly dissimilar to the core of brands for extending business scale. Further, we subdivided different empathy characters of consumer groups to investigate these issues. Thus, the findings not only serve to understand the cognitive mechanisms of different consumers how to process brand association messages, but in an effort to give valuable guidance for market managers in distant extension strategies.

However, there are some limitations in this study. First, we only selected the empathy level as the individual difference examining factor in the experiment. Besides, demographic profiling factors, such as gender, should be seen as a proxy variable. A prior research has pointed out that women commonly score higher on the empathy level than men did, and the brand perception (e.g., perceived warmth or competence) was distinct between male and female subjects (Jaffee and Hyde, 2000; Xue et al., 2020). Future studies could consider the gender factor to examine the behavioral and neural differences when evaluating distant extensions under different brand association conditions. Second, considering the limited environmental and rigorous stimuli design requirements of ERP experiments, the current experiment used text presentation formation as stimuli similar to a previous brand extension research. Therefore, the brand association and extension evaluation scenario in the experiment was not perfectly close to reality. Indeed, future work can alter the presentation of stimulus materials and adopt other neuroscientific tools, such as FMRI, to overcome the issue.

## Conclusion

The current study focused on individual empathy traits and brand associations, followed by the neural mechanism of CSR and CC associations affecting distant extension products in the high and low empathy groups. In the early stage of distant extension evaluation, the LE group elicited larger N2 amplitude than the HE group, which is a conflict detection processing. In addition, an augment N2 was found under CSR than CC associations in the LE group, reflecting that individuals with LE levels might allocate more cognitive resources to CSR than CC information. At the later stage, the HE group produced more positive P3 amplitude than the LE group. In addition, a larger P3 was observed under CSR than CC associations in the HE group. The results indicate that individuals with high empathy levels might engage the motivational salience and improve the helping willingness when evaluating the distant brand extension, especially under CSR associations. These findings suggest that brand association types (CSR and CC) influence distinct stages of mental processing of consumers with different empathy levels toward the distant extension evaluation.

## Data Availability Statement

The original contributions presented in the study are included in the article/Supplementary Material, further inquiries can be directed to the corresponding author/s.

## Ethics Statement

The studies involving human participants were reviewed and approved by the institutional review board of Yanshan University. The patients/participants provided their written informed consent to participate in this study.

## Author Contributions

ZS, CL, RS, and KJ contributed to the construction and design of the research. CL and RS performed the experiment. CL analyzed the data and wrote the manuscript. ZS, KJ, and RS reviewed the article. All authors contributed to the article and approved the submitted version.

## Conflict of Interest

The authors declare that the research was conducted in the absence of any commercial or financial relationships that could be construed as a potential conflict of interest.

## Publisher’s Note

All claims expressed in this article are solely those of the authors and do not necessarily represent those of their affiliated organizations, or those of the publisher, the editors and the reviewers. Any product that may be evaluated in this article, or claim that may be made by its manufacturer, is not guaranteed or endorsed by the publisher.
